# Smart Modification on Magnetic Nanoparticles Dramatically Enhances Their Therapeutic Properties

**DOI:** 10.3390/cancers13164095

**Published:** 2021-08-14

**Authors:** Nuria Lafuente-Gómez, Paula Milán-Rois, David García-Soriano, Yurena Luengo, Marco Cordani, Hernán Alarcón-Iniesta, Gorka Salas, Álvaro Somoza

**Affiliations:** 1 Instituto Madrileño de Estudios Avanzados en Nanociencia (IMDEA Nanociencia), Faraday 9, 28049 Madrid, Spain; nuria.lafuente@imdea.org (N.L.-G.); paula.milan@imdea.org (P.M.-R.); david.garcia.soriano@imdea.org (D.G.-S.); yurena.luengo@imdea.org (Y.L.); marco.cordani@imdea.org (M.C.); hernan.alarcon@imdea.org (H.A.-I.); gorka.salas@imdea.org (G.S.); 2 Unidad Asociada al Centro Nacional de Biotecnología (CSIC), Darwin 3, 28049 Madrid, Spain

**Keywords:** nanomedicine, magnetic nanoparticles, drug delivery, magnetic hyperthermia

## Abstract

**Simple Summary:**

In this work, a smart gemcitabine delivery system based on magnetic nanoparticles (MNP) is proposed. Gemcitabine (GEM) is a chemotherapeutic agent usually employed as monotherapy for the treatment of pancreatic cancer. Unfortunately, this drug presents short half-life and high toxicity in non-tumoral tissues. Thus, new efficient drug delivery systems are needed. In this regard, we modified MNP to attach this drug via disulfide bonds (MNP-GEM) to promote the selective release of GEM in pancreatic cancer cells, and the great potential of our proposed nanocarrier for biomedical applications is broadly assessed. Remarkably, this modification has proved to prevent the unspecific binding of proteins, reduced the cytotoxic effect of the drug in non-cancerous cells, improved the internalization in pancreatic cancer cells, and its activity was synergistically enhanced in combination with magnetic hyperthermia.

**Abstract:**

Magnetic nanoparticles (MNP) are employed as nanocarriers and in magnetic hyperthermia (MH) for the treatment of cancers. Herein, a smart drug delivery system composed of MNP functionalized with the cytotoxic drug gemcitabine (MNP-GEM) has been thoroughly evaluated. The linker employed is based on a disulfide bond and allows the controlled release of GEM under a highly reducing environment, which is frequently present in the cytoplasm of tumor cells. The stability, MH, and the interaction with plasma proteins of the nanoparticles are evaluated, highlighting their great potential for biological applications. Their cytotoxicity is assessed in three pancreatic cancer cell lines with different sensitivity to GEM, including the generation of reactive oxygen species (ROS), the effects on the cell cycle, and the mechanisms of cell death involved. Remarkably, the proposed nanocarrier is better internalized than unmodified nanoparticles, and it is particularly effective in PANC-1 cells, resistant to GEM, but not in non-tumoral keratinocytes. Additionally, its combination with MH produces a synergistic cytotoxic effect in all cancer cell lines tested. In conclusion, MNP-GEM presents a promising potential for treating pancreatic cancer, due to multiple parameters, such as reduced binding to plasma proteins, increased internalization, and synergistic activity when combined with MH.

## 1. Introduction

Cancer is among the leading causes of death worldwide [1] and, specifically, pancreatic ductal adenocarcinoma (PDAC) is one of the most lethal, mainly due to the poor prognosis and late diagnosis, as well as resistance to current drug therapies [2]. Gemcitabine, a deoxycytidine analog, is the gold-standard chemotherapy issued as a monotherapy [3,4]. Unfortunately, this drug presents low efficacy due to its short half-life, low bioavailability, and the development of drug resistance. That is why it needs frequent administration with a high dose, ending with severe systemic toxicity of healthy cells [4].

In the last decades, nanotechnology has received tremendous attention for its applications to medicine. Many scientists are focusing their research effort on designing stimuli-responsive or smart nanocarriers for biomedical applications, including cancer diagnosis and treatment [5,6]. These devices may be sensitive to specific endogenous stimuli (e.g., redox potential, changes in pH, the concentration of specific analytes) or exogenous stimuli (e.g., magnetic field, light, temperature, ultrasounds), allowing for the spatial and temporal control of the release of bioactive compounds [7]. Hence, using smart nanoparticles could overcome the shortcomings of traditional chemotherapy, such as the lack of specificity and side effects [8,9]. In this sense, magnetic nanoparticles have been widely studied [10,11,12,13]. They can be used as a diagnostic tool in magnetic resonance imaging (MRI), promote cancer cell death via magnetic hyperthermia (MH), which involves the generation of heat when an alternating magnetic field (AMF) is applied [10,14,15,16], and also as carriers of anticancer drugs [17,18,19]. Despite all the efforts done to understand the mechanisms involved in cell death and to improve the therapeutic efficacy of MNP [10,13], a comprehensive report on cell–nanoparticle interactions is needed.

In this study, we proposed magnetic nanoparticles that consist of a maghemite core coated with the polysaccharide dextran (MNP). In this regard, these nanostructures have been functionalized with gemcitabine (MNP-GEM) by employing a linker containing a disulfide bond. Interestingly, this linker was designed to release the drug without any chemical modification [20]. This process primarily occurs inside the cells, where glutathione (GSH) concentration is in the millimolar range (0.5–10 mM). On the other hand, GSH concentration in blood plasma and the extracellular medium is in the micromolar range [20,21]. This difference ensures the integrity of the system in the blood for several hours. Additionally, tumor tissues present higher levels of glutathione than normal tissues [22], which can be exploited to better control the GEM release at the target tissue. This nanosystem was fully characterized, and its stability was assessed in different media. Moreover, we studied the heating efficiency of our system with the application of an AMF to produce MH [10]. Furthermore, to better assess the therapeutic potential of our MNP-GEM, we carried out cell viability tests, cell cycle analyses, and internalization studies in three different pancreatic cell lines, PANC-1, BxPC-3 and MIA Paca-2, with different sensitivity to GEM. The biocompatibility of the system was also tested in the non-tumoral cell line HaCaT, and the efficacy of the system was also evaluated in the breast cancer cell line MCF-7. Additionally, cell death mechanisms, such as necrosis, apoptosis, reactive oxygen species (ROS) production, and autophagy, were also assessed. Finally, we evaluated the cell viability in all pancreatic cell lines treated with MNP and MNP-GEM after applying an AMF to determine the effect of MH in cells. Thus, these studies will provide insights on therapies based on MH using functionalized nanoparticles to treat pancreatic cancer, both sensitive and resistant to chemotherapeutics. 

## 2. Materials and Methods

A detailed description of the materials used can be found in the Supplementary Materials.

### 2.1. Synthesis of MNP

The γ-Fe_2_O_3_ cores were synthesized by coprecipitation [23], followed by an optimized acid treatment [24]. Briefly, NH_4_OH 25% (75 mL) was added at 0.1 mL/s to an aqueous solution of FeCl_2_ 0.175 M and FeCl_3_ 0.334 M (500 mL) under vigorous stirring. After 5 min, the reaction was heated to 90 °C for 3 h and stirred overnight at room temperature. Then, the precipitate was isolated by magnetic decantation; HNO_3_ 2 M (300 mL) was added, and the stirring was maintained for 15 min. After that, HNO_3_ was removed by magnetic decantation, and Fe(NO_3_)_3_ 1 M (75 mL) and distilled water (130 mL) were added. The mixture was boiled and stirred for 30 min. Then, the solution was cooled down, the supernatant was removed by magnetic decantation, and HNO_3_ 2 M (300 mL) was added and stirred for 15 min. The γ-Fe_2_O_3_ cores were washed with water and concentrated in a rotary evaporator. 

For surface modification with Dextran, a slightly modified published procedure was used [25]. A dispersion of particles with 228 mg Fe_2_O_3_/L in NaOH 0.8 M (1.6 mL) was added dropwise into a solution of NaOH 0.5 M (2.5 mL) with Dextran 40 kDa (200 mg). The mixture was sonicated for 6 h under refrigeration. Then, MNP were dialyzed and the pH was adjusted to 7.

### 2.2. Covalent Attachment of Gemcitabine on MNP (MNP-GEM)

MNP-GEM were prepared based on previous reports with modifications [20]. Firstly, to MNP at 2 mg Fe/mL (1 mL) were added 600 μmol of EDC/g Fe (20 μL 120 mM) and 300 μmol of NHS/g Fe (20 μL 60 mM), which was then stirred overnight. The MNP were washed by 3 cycles of centrifugation and redispersion. Then, 200 μmol of cysteamine hydrochloride/g Fe (20 μL 40 mM), neutralized by 200 μmol of NaOH/g Fe (20 μL 40 mM), were added. After 16 h continuous stirring, the MNP were washed again and mixed with 50 μmol of Gem-s-s-Pyr/g Fe (200 μL 500 μM in DMF) (synthesis procedure of GEM-s-s-Pyr described in Figure S1) for 16 h under continuous stirring. To quantify the amount of GEM incorporated in MNP, brine (20 μL) was added to MNP-GEM to eliminate the possible electrostatically immobilized GEM-s-s-Pyr, and the sample was centrifuged for 40 min at 19,600× *g*. From the collected supernatants, the GEM incorporated was determined by quantification of the 2-pyridinethione released (λ_max_ 343 nm, ε_343nm_ 8080 L-mol^−^^1^·cm^−^^1^). Finally, MNP-GEM were redispersed in 1 mL of water.

### 2.3. In Vitro Drug Release Studies

The release of the gemcitabine from MNP-GEM at 2 mg Fe/mL was carried out under physiological conditions (37 °C and PBS, pH 7.4) and two different concentrations (1 µM or 1 mM) of the reducing agent 1,4-dithiothreitol (DTT). The amount of gemcitabine (GEM) released was determined after 8 h by measuring the absorbance of the sample at 270 nm in a Synergy H4 microplate reader. The percentage of GEM release was calculated from a standard calibration curve of free drug solution (Figure S12). The blank solutions were composed by MNP with the DTT concentrations tested.

### 2.4. Characterization of MNP and MNP-GEM

Transmission electron microscopy (TEM) images were collected to determine the particle size distribution and shape (JEOL JEM 1010), operating at 80 kV at CBMSO-CSIC). Inductively coupled plasma optical emission spectrometry (ICP-OES) was used to determine the iron concentration in the nanoparticles’ dispersions before their functionalization (Perkin Elmer Optima 2100 DV at ICMM-CSIC). The hydrodynamic diameter and zeta potential were obtained in a Zetasizer (Nano-ZS device, Malvern Instruments). The stability of the magnetic nanoparticles was evaluated in three different media (water, PBS, and DMEM supplemented with 10% FBS) by measuring the hydrodynamic size. X-ray diffraction (XRD) patterns of the lyophilized samples were acquired on a Bruker SOL-X D8 Advance system (with Cu Kα radiation, scan angle 2θ = 20°–80° at a 0.04 scan step, using a D5000 diffractometer equipped with a secondary monochromator). Simultaneous thermogravimetric/differential thermal analyses (TGA/DTA) were done in a TA Instruments TGA 500, with a heating rate of 10 °C min^−^^1^, in air atmosphere from room temperature to 900 °C. The magnetic characterization was carried out in a vibrating sample magnetometer (VSM; MLVSM9 Mag Lab 2 T, Oxford Instrument). Approximately 3 mg of nanoparticles were compressed into a cellulose capsule with a cleaning cotton. After saturating the samples in a field of 2 T, the sample magnetization (M (emu)) vs. applied magnetic field (H (tesla)) curves were acquired at room temperature. 

### 2.5. Evaluation of the Protein Binding

MNP and MNP-GEM were added to 1 mg/mL bovine serum albumin (BSA) solution at different concentrations (from 1 to 125 × 10^−^^5^ M) and incubated for 24 h. The binding parameters were evaluated by selectively exciting the tryptophan residues of BSA (λ_ex_ 295 nm, λ_em_ 340). The measurements were carried out at 26.5 °C and 37 °C in a Synergy H4 microplate reader. The possible quenching mechanism was analyzed by the Stern–Volmer equation, and the nature of the binding forces was determined by evaluating the thermodynamic parameters using the Van’t Hoff equation [26,27]. The detailed formulas can be found in the Supporting Information.

### 2.6. Magnetic Hyperthermia Evaluation in Solution

The magnetic hyperthermia (MH) produced by MNP and MNP-GEM dispersed in water, DMEM, and RPMI at 0.5 mg Fe/mL and 0.1 mg Fe/mL was evaluated when an alternating magnetic field (AMF) was applied for 20 min (frequency 202 KHz, amplitude 29.9 mT) in the AC field applicator DM100 (Nanoscale Biomagnetics) within a working space thermally insulated at 37 °C. The temperature of the colloids was measured using an optic fiber sensor. To obtain the data of specific absorption rates (SAR, W/g Fe), Equation (1) is applied.

(1) SAR=(mwaterCwatermFe)× (ΔTΔt)

where C_water_ is the water specific heat (4.185 J/g K^−^^1^), and m_Fe_ is the mass of iron diluted in the sample. To obtain the value of ∆T/∆t a linear fit of the data (temperature vs. time) was performed in the initial time interval (time interval 30–60 s) once the alternating magnetic field was turned on. 

### 2.7. Cell Viability Assays

Cells were seeded in 24-well plates and treated at 60% confluency for 24 h. Then, cells were washed with PBS 1X to remove the not internalized drug or nanoparticles, and fresh medium was added. The alamarBlue Viability assays were done the following two days (48 and 72 h after treatment). For the evaluation of MNP as drug delivery systems in combination with MH, cells were seeded in culture dishes with 4 wells for self-insertion, attached to cell culture dishes of 35 mm, and treated at 60% confluency for 24 h. Then, the AMF was applied as mentioned before, cells were washed, and AlamarBlue assays were done 48 h and 72 h after treatment. To perform the AlamarBlue assays, a stock solution of resazurin sodium salt (1% *v*/*v*) in complete medium was added to the cells. After 3 h at 37 °C in the incubator, the fluorescence was measured at 25 °C (λ_ex_ 550 nm, λ_em_ 590 nm). The fluorescent intensity measurements were processed using Equation (2), wherein the positive control corresponds with untreated cells, and the negative control was a resazurin solution in complete medium without cells.

(2) $$\%\;\mathrm{Cell}\;\mathrm{viability}=\frac{\mathrm{Sample}\;\mathrm{data}-\mathrm{Negative}\;\mathrm{Control}}{\mathrm{Positive}\;\mathrm{Control}-\mathrm{Negative}\;\mathrm{Control}}\times 100$$

In PANC-1 and HaCaT cells, the concentrations tested were: MNP 0.1, 0.5 and 2 mg Fe/mL; GEM 4.5, 22.5 and 90 µM; MNP-GEM 0.1 mg Fe/mL 4.5 µM, 0.5 mg Fe/mL 22.5 µM, 2 mg Fe/mL 90 µM.

In BxPC-3, MiaPaca-2 and MCF-7 cells, the concentrations tested were: MNP 0.1 mg Fe/mL; GEM 4.5 µM; MNP-GEM 0.1 mg Fe/mL 4.5 µM.

### 2.8. MNP Cellular Uptake Studies

The internalization of MNP and MNP-GEM was evaluated using three different methods. MNP or MNP-GEM were incubated for 24 h in P6 plates with cancer cells at 60% confluency. Then, cells were washed with PBS 1× to remove the not internalized nanoparticles and fresh medium was added. The internalization assays were performed the following day, 48 h after treatment.

- Prussian blue staining [28]. Briefly, cells were fixed in ice-cold methanol for 5 min. Then, the cells were stained with an equal volume of 2% HCl and 2% potassium ferrocyanide trihydrate for 15 min and counterstained with 0.5% neutral red for 3 min. Finally, the preparations were mounted in DePeX and visualized in a LeicaDMI300 B optical microscope.
- Colorimetric ferrozine-based assay [29]. Briefly, aliquots of cell lysates in 50 mM NaOH (100 μL) were mixed with equal volumes of 10 mM HCl and an iron-releasing agent (1.4 M HCl and 4.5% p/p KMnO4 in water). The mixtures were incubated for 2 h at 60 °C and cooled to room temperature. Then, the iron-detection reagent (30 μL) was added (6.5 mM ferrozine, 6.5 mM neocuproine, 2.5 M ammonium acetate, and 1 M ascorbic acid in water). After 30 min, the absorbance at 565 nm was measured on a microplate reader. The same procedure was used for the calibration line with our MNP.
- TEM images in cell culture: Cells were fixed with a mixture of paraformaldehyde (4%) and glutaraldehyde (2%). Then, the electronic service of the Molecular Biology Severo Ochoa Center examined the samples for the posterior visualization in a transmission electron microscope JEOL JEM 1010.

To elucidate the internalization pathways of the nanoparticles, cells were seeded in P6 wells and treated at 60% confluency with endocytosis inhibitors for 2 h (Table S1). Then, cells were washed with PBS 1×, and nanoparticles were incubated for 4 h. After that time, the colorimetric ferrozine-based assay was carried out. The data obtained was normalized vs. the control (cells treated with MNP and MNP-GEM without inhibitors) and represented as % of internalized Fe.

### 2.9. Cell Cycle Analysis

Cells were seeded in 6-well plates and treated at 60% confluency for 24 h. Then, cells were washed with PBS 1× to remove the not internalized drug or nanoparticles, and fresh medium was added. The following day, 48 h after treatment, the samples were trypsinized, washed with PBS 1×, and centrifuged at 177× *g* for 5 min. Cells were fixed in cold ethanol 70% for 15 min, and then centrifuged at 177× *g* for 15 min to completely remove the ethanol. Each sample was treated with 10 µg RNAsa A and 20 µg PI for a total volume of 500 µL in PBS. Cell cycle analysis was performed in a Beckman Coulter Cytomics 500 Flow Cytometer using 20,000 cells. The acquired data was analyzed with Flowing Software. These experiments were performed in the Flow Cytometry Service at the CNB-CSIC.

### 2.10. Measurement of Intracellular ROS

Cells were seeded in 96-well plates and treated at 60% confluency for 24 h. Then, they were washed with PBS 1X and incubated with 5 μM diacetylated 2′,7′-dichlorofluorescein (DCF-DA) probe for 15 min at 37 °C. Then, cells were washed again, and DCF-DA fluorescence was measured in the Synergy H4 Hybrid multimode plate reader (λ_exc_ 485 nm, λ_em_ 535 nm). Values were normalized on alamarBlue viability assay. It was repeated 48 and 72 h after treatment.

### 2.11. Monodansylcadaverine Staining and Autophagosome Detection

Cells were seeded in 96-well plates and treated at 60% confluency for 24 h. Then, they were washed twice with 1× PBS and incubated for 15 min in culture medium with 50 μM monodansylcadaverine (MDC) at 37 °C. Then, cells were washed again, and fluorescence was measured in the Synergy H4 Hybrid multimode plate reader. Values were normalized on alamarBlue viability assay. It was repeated 48 and 72 h after treatment.

### 2.12. Necrosis/Apoptosis Assay

Cells were seeded in 6-well plates and treated at 60% confluency for 24 h. Then, the supernatants were taken, and the cells were trypsinized. The obtained cells were centrifuged at 177× *g* for 5 min and resuspended in 100 µL binding buffer 1×. Then, 10 µL annexin V 1× were added and incubated 15 min at 4 °C in darkness. After that, 380 µL of the binding buffer 1× were added to the samples. Finally, 10 µL propidium iodide 1 mg/mL was just added before analysis acquisition in a Beckman Coulter Cytomics 500 Flow Cytometer using 20,000 cells. These experiments were performed in the Flow Cytometry Service at the CNB-CSIC.

### 2.13. Western Blot

To study the effect of the treatments in cyclin E, 250,000 cells per P6 well were seeded, and on the next day, cells were treated for 24 h. The following day, cells were lysed with lysis buffer plus protein inhibitors (10 mM Tris-HCl pH 7.5, 5 mM EDTA, 150 mM sodium chloride, 10% glycerol, 0.5% Triton X-100, 50 mM sodium fluoride, 30 mM sodium pyrophosphate, 1 mM sodium orthovanadate, and 1 mM phenylmethylsulfonyl) by scrapping the surface of the wells and incubating cell extracts for 30 min at 4 °C in a tube rotator. The total cell lysate was cleared by centrifugation (15 min at 16,100× *g*, 4 °C), and supernatants were stored at −80 °C. To study the effect of GEM in the phosphorylation of HSP-27, 550,000 cells per P6 well were seeded. After 24 h, cells were treated for 4 h, and they were lysed as described above. The total protein amount was quantified by Bradford assay (Bio-Rad, Richmond, CA, USA). Twenty μg of protein samples were separated on 15% SDS-polyacrylamide gels under reducing conditions and transferred to 0.45 μm nitrocellulose membrane (GE Healthcare Life science, Piscataway, NJ, USA). After incubating the membranes with 5% BSA in TBS-T (0.1% Tween-Tris buffered saline) for 1 h at room temperature, the blots were incubated overnight at 4 °C with the corresponding first antibody solution (anti-cyclin E 1:500, anti-HSP27 1:5000, anti-phospho HSP27 1:2500, or anti-GADPH 1:500). in 3% BSA in TBS-T. After three washes with TBS-T, blots were incubated with peroxidase-labeled anti-mouse (1:5000) with 3% BSA in TBS-T blocking solution for 1 h at room temperature. Then, they were washed again and membrane-bound antibody was detected with enhanced chemiluminescence detection reagent (Bio-Rad). Densitometry analysis was performed using Fiji software (ImageJ).

### 2.14. Statistical Analysis

Microsoft Excel (Office 365) was used for graphical representations and R Project for Statistical Computing (R-3.2.5) software (R Development Core Team) for statistical analysis. One-way ANOVA was used to compare the mean value of each condition vs. control, and a Student’s *t*-test was used when only two conditions need to be compared. The threshold for significance was *p* = 0.05 and *p* < 0.05 (*), *p* < 0.01 (**), and *p* < 0.001 (***). Tukey’s test was performed to compare the mean values by pairs when a statistical difference was observed.

## 3. Results

### 3.1. Synthesis and Characterization of MNP and MNP-GEM

**Figure 1:**
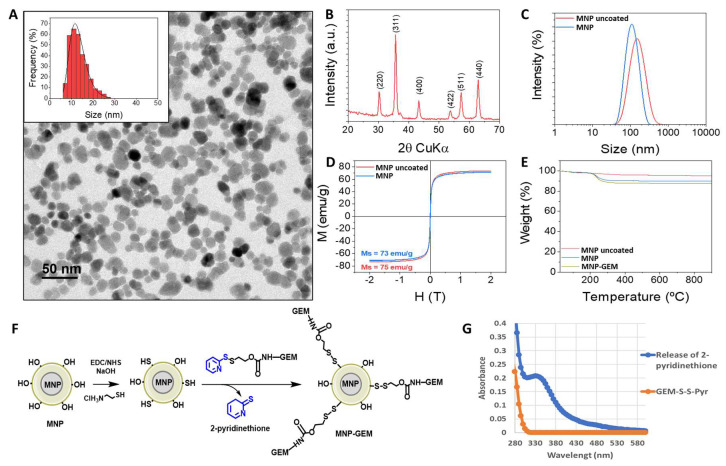
(**A**) TEM micrographs and size distributions (inset) of 14 nm γ-Fe_2_O_3_ cores (MNP uncoated); (**B**) X-ray diffraction pattern of MNP uncoated prepared by coprecipitation; (hkl) indices corresponding to a maghemite phase are included for peak identification; (**C**) Hydrodynamic size distribution of MNP uncoated and after dextran coating (MNP); (**D**) Magnetization curves at room temperature of MNP uncoated and MNP; (**E**) Thermogravimetric analyses of MNP uncoated, MNP, and MNP-GEM; (**F**) General scheme of functionalization of MNP with GEM (MNP-GEM) via disulfide bonds; (**G**) UV spectra of GEM immobilization. The absorbance at λ_343_ corresponds to the 2-pyridinethione released during the functionalization of MNP-GEM.

Magnetic nanoparticles composed of a core of maghemite (γ-Fe_2_O_3_) with a mean size of 14 nm (MNP uncoated) were prepared by coprecipitation (Figure 1A,B) [23,24]. Then, they were successfully coated with dextran (MNP) [25], leading to a stable colloidal formulation in water with a hydrodynamic size of ca. 100 nm (Figure 1C). MNP displayed a superparamagnetic behavior with saturation magnetization values around 72 emu/g, close to the value of bulk maghemite (Figure 1D). Later on, MNP were modified with the drug (MNP-GEM) (Figure 1F, synthetic procedure of the linker described in Figure S1), and the amount of GEM attached (45 μmol GEM/g Fe, 90% yield, [Fe] 2 mg/mL) was determined by quantifying the 2-pyridinethione released during the conjugation process (Figure 1G). Finally, the nanoparticles were studied by thermogravimetry, revealing a pattern consistent with the increasing amount of organic matter due to the dextran and the functionalization with GEM (Figure 1E).

The colloidal stability in water was evaluated by measuring the hydrodynamic diameter and zeta potential of MNP (101 nm, PDI 0.15; −12.3 mV) and MNP-GEM (102.8 nm, PDI 0.174; +15.1 mV). Moreover, the nanoparticles presented good stability in water, PBS and DMEM supplemented with 10% FBS for seven days (Figure S2), but MNP-GEM seems to start aggregating in PBS after 2 days.

### 3.2. Synthesis and Characterization of MNP and MNP-GEM

The release of the drug from MNP-GEM was evaluated. After 8 h, the results showed that 100% of the drug was released from MNP-GEM under intracellular tumor cells conditions (1 mM DTT), whereas only 10% was released from MNP-GEM in the environment mimicking blood plasma and extracellular medium (1 µM DTT) (Figure S3). 

### 3.3. Evaluation of the Protein Binding

We evaluated the changes in the intrinsic fluorescence intensity of albumin, the main protein in the bloodstream, in the presence of MNP and MNP-GEM, which is considered a useful method to determine nanoparticle–protein binding parameters [26,27]. The fluorescence intensity of albumin gradually decreased when the MNP or MNP-GEM concentration increased, due to the interactions that exist between them (Figure S4). The type of quenching that occurs can be elucidated by the analysis of bimolecular quenching constant values (k_q_) and the Stern–Volmer quenching constant (k_sv_) (Table 1) obtained using the Stern–Volmer equation (Equation (S1)).

The value of k_q_ is greater than the limiting diffusion rate constant of the biomolecules (2.0 × 10^10^ M^−^^1^·s^−^^1^), so the static quenching mechanism could be predominant [26]. Moreover, higher temperature results in a smaller value of k_sv_, which confirms the static quenching [26]. In this case, the k_sv_ is considered the binding constant (k_b_) [26,27]. These results indicate a moderate affinity (k_sv_, k_b_ are in the order of 3 and 4) and, what is more interesting, the functionalization of MNP with GEM decreases the binding constants. Moreover, to elucidate the nature of the binding force, the thermodynamic parameters of the complexes were calculated (Table S2) based on the Van’t Hoff equation and thermodynamic equations (Equations (S2)–(S4)). According to the data, the binding is spontaneous (∆G < 0) [30] and mainly electrostatic (∆H < 0 and ∆S > 0) [26].

### 3.4. Magnetic Hyperthermia Evaluation in Solution

The efficiency of MH therapy is mainly determined by the specific absorption rates (SAR) values of MNP, but higher SAR values do not mean higher temperatures [31,32]. The increase in temperature might be affected by the material employed, the dose, and the media [33]. Thus, we compared the generation of MH produced by MNP and MNP-GEM at two concentrations (0.5 and 0.1 mg Fe/mL) in water, DMEM and RPMI (Table 2 and Figure S5). We observed significant differences in the SAR and temperature obtained, probably due to the interactions between the surface of the nanoparticles and the media. In this case, higher SAR values were observed at 0.1 mg Fe/mL than at 0.5 mg/mL, due to the non-monotonic dependence of the SAR with the concentration [33,34]. Despite that, higher temperatures were usually reached at higher concentrations as expected for higher MNP loads. It is also worth noting that these values also change depending on the media.

### 3.5. Cell Viability Assays

To assess our therapeutic system, we employed three pancreatic cell lines (PANC-1, BxPC-3, and MIA Paca-2) and a no-tumoral cell line (HaCaT). 

**Figure 2:**
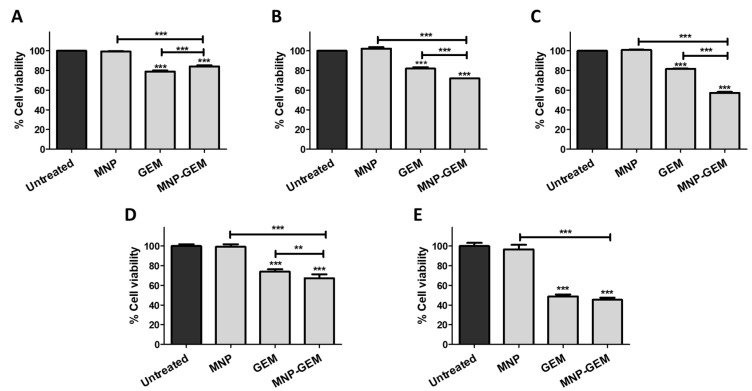
Cell viability assays 72 h after treatment in: (**A**–**C**) PANC-1; (**D**) BxPC-3; (**E**) MIA Paca-2. Conditions tested (**A**)**,** (**D**,**E**) MNP 0.1 mg Fe/mL, GEM 4.5 µM, MNP-GEM 0.1 mg Fe/mL 4.5 µM. (**B**): MNP 0.5 mg Fe/mL, GEM 22.5 µM, MNP-GEM 0.5 mg Fe/mL 22.5 µM. (**C**): MNP 2 mg Fe/mL, GEM 90 µM, MNP-GEM 2 mg Fe/mL 90 µM. Data represent means ± SD. Statistical analysis was performed using a one-way ANOVA test (each group vs. control). ** *p* < 0.01, *** *p* < 0.001.

Firstly, the results in the gemcitabine-resistant cell line PANC-1 (Figure 2A–C, Figure S6A–C) showed that MNP-GEM had a dose-dependent cytotoxic effect, especially relevant 72 h after treatment. What is more, after that time, MNP-GEM reduced the cell viability more than the free drug at higher concentrations (22.5 and 90 µM). Secondly, MNP-GEM were also tested in gemcitabine-sensitive BxPC-3 and MIA Paca-2 cells (Figure 2D,E, Figure S6D,E). In the case of BxPC-3 cells, the reduction in cell viability after 48 h of treatment with GEM or MNP-GEM was similar (Figure S6D), but after 72 h of treatment, MNP-GEM was slightly more effective (*p* < 0.001) (Figure 2D). On the other hand, in MIA Paca-2 there were no differences among the cytotoxic effect of GEM and MNP-GEM (Figure 2E and Figure S6E). 

In a similar way, the nanostructures were tested in a non-pancreatic cell line, MCF-7, where the effect observed with MNP-GEM was similar to the one obtained with GEM (Figure S7A,B). Considering the efficacy of MNP-GEM, this approach could be applied to different cancer models.

Finally, the same conditions tested in gemcitabine-resistant cell line PANC-1 were also evaluated in non-tumoral cells HaCaT. Remarkably, MNP-GEM are less toxic than GEM at all concentrations and times tested (Figure S7C–H). What is more, only at the highest concentration (2 mg Fe/mL 90 μM GEM) did our proposed nanocarrier presents cytotoxicity against no-tumoral cells. 

Based on these results, the concentration 0.5 mg Fe/mL was selected for subsequent studies in PANC-1 cells since it is the lowest concentration tested where MNP-GEM presents higher activity than the free drug. On the other hand, 0.1 mg Fe/mL were employed for the studies in BxPC-3 and MIA Paca-2.

### 3.6. Cell Cycle Analysis

**Figure 3:**
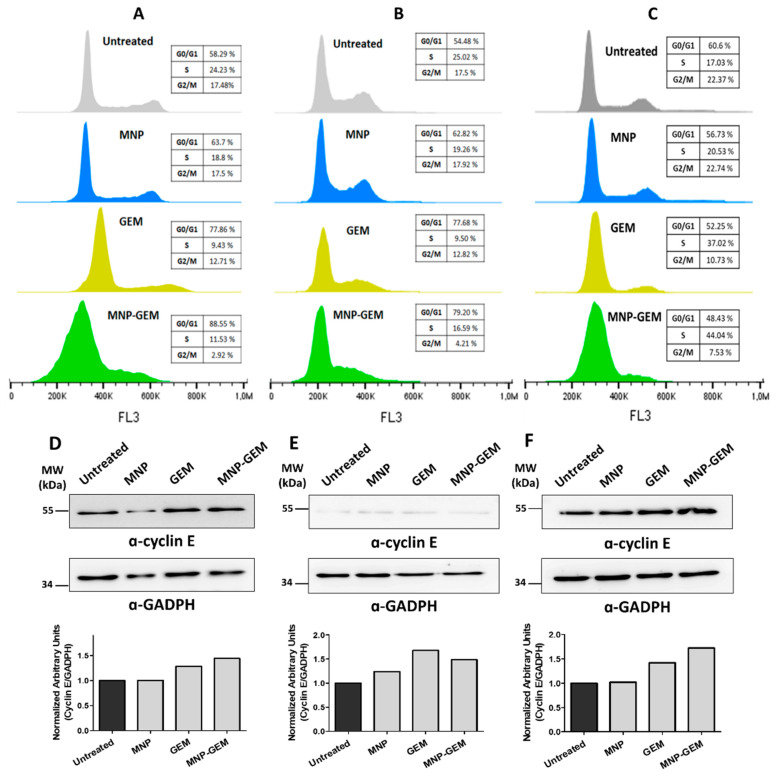
Flow cytometry analysis of the cell cycle 48 h after treatment in: (**A**) PANC-1; (**B**) BxPC-3; (**C**) MIA Paca-2. Analysis of cyclin E protein levels in: (**D**) PANC-1; (**E**) BxPC-3; (**F**), and MIA Paca-2. Densitometry analysis plots show arbitrary units calculated as cyclin E signal normalized to GADPH signal. Conditions for (**A**,**D**): MNP 0.5 mg Fe/mL, GEM 22.5 µM, MNP-GEM 0.5 mg Fe/mL 22.5 µM; (**B**,**C**,**E**,**F**): MNP 0.1 mg Fe/mL, GEM 4.5 µM, MNP-GEM 0.1 mg Fe/mL 4.5 µM.

To know the effect of MNP, GEM and MNP-GEM on cell cycle distribution, flow cytometry analysis was performed. Interestingly, in all cell lines tested, MNP had no significant effect on the cell cycle (Figure 3). Regarding the effect of GEM alone in pancreatic cancer cell lines, a reduction in G2/M phase and a cell cycle arrest in G0/G1 phase was observed. Remarkably, it was even more pronounced using MNP-GEM both in PANC-1 (Figure 3A) and BxPC-3 cells (Figure 3B). However, in MIA Paca-2 cells, this arrest seemed to be produced in S phase (Figure 3C). 

To further assess the implications of MNP-GEM in the arrest of the cell cycle observed, the levels of cyclin E, which is involved in the G1-S transition [35,36], were studied by Western blot in PANC-1 (Figure 3D), BxPC-3 (Figure 3E), and MIA Paca-2 (Figure 3F). According to our data, cyclin E is more accumulated in GEM and MNP-GEM treated cells (Figure 3D–F).

### 3.7. MNP Cellular Uptake Studies

**Figure 4:**
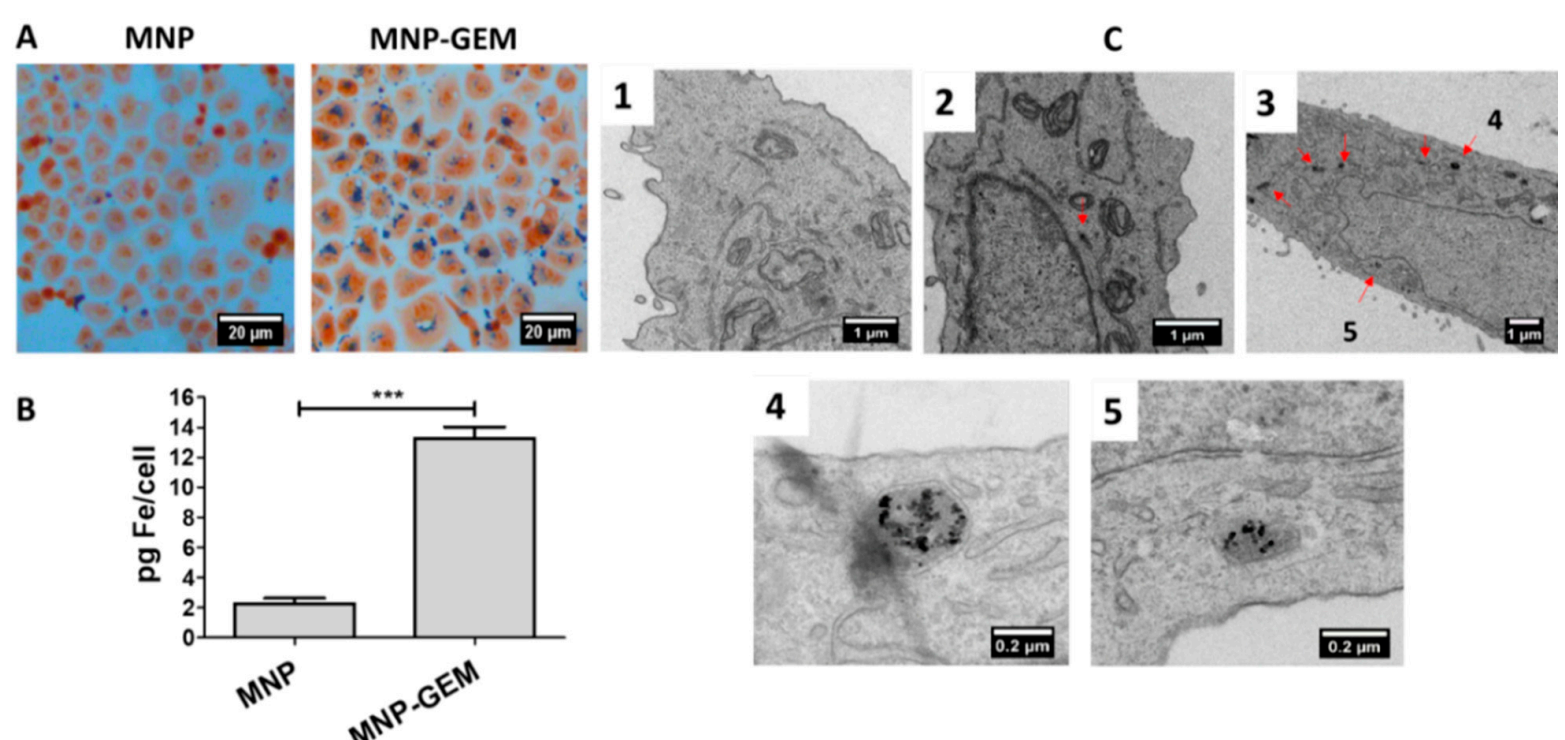
Internalization study of MNP and MNP-GEM in PANC-1 cells at 0.5 mg Fe/mL. (**A**) Prussian Blue staining. (**B**) Ferrozine assay (mean values ± SD, Student’s *t*-test, *** *p* < 0.001). (**C**) TEM images (C1. Untreated; C2. MNP; C3, C4 and C5. MNP-GEM).

The internalization of MNP into cells plays a crucial role in their safety and efficacy [37]. Thus, the internalization and the mechanism of endocytosis involved were studied. Particularly, the intracellular uptake of MNP and MNP-GEM (0.5 mg Fe/mL) was studied in PANC-1 cells using Prussian blue staining, colorimetric ferrozine assay, and TEM images (Figure 4).

By Prussian blue staining, we could observe that MNP-GEM were internalized better than MNP, which seemed to be localized in the cytoplasm and did not enter the nucleus (Figure 4A). Additionally, the colorimetric ferrozine-based assay was used to estimate iron concentration inside cells. It was established that the amount of iron per cell in PANC-1 treated with MNP-GEM was almost six times higher than in the cells treated MNP (Figure 4B). TEM images confirm that MNP are less internalized than MNP-GEM and show that the nanoparticles were accumulated in cytoplasmic vesicles (Figure 4C).

Similar results were obtained in BxPC-3 (Figure S8A,B) and MIA Paca-2 (Figure S9C,D) when cells were treated with the nanoparticles (0.1 mg Fe/mL). Prussian-blue-stained cells suggested that the nanoparticles were localized in the cytoplasm (Figure S8A,C). Particularly, in BxPC-3 cells, the internalization of MNP-GEM was almost 8 times higher than MNP (Figure S8B), and, in MIA Paca-2, it was 7 times higher, according to the data obtained from ferrozine assay (Figure S8D).

To elucidate the internalization pathways involved, classic endocytosis inhibitors were selected (Table S2) [38,39,40], and a ferrozine assay was done to determine nanoparticles’ internalization (Figure S9). Data suggest that there are no differences in the mechanisms of internalization among MNP and MNP-GEM, but there are differences among cell lines. PANC-1 cells internalized the nanoparticles via caveolin-mediated endocytosis, macropinocytosis, and phagocytosis, and in BXPC-3 and MIA Paca-2 cells, clathrin-mediated endocytosis is also involved.

### 3.8. Measurement of Intracellular ROS

ROS production is strongly associated with the physiology of cancer [41], and most of the current chemotherapy treatments exploit this phenomenon to induce cancer cell death [42,43].

**Figure 5:**
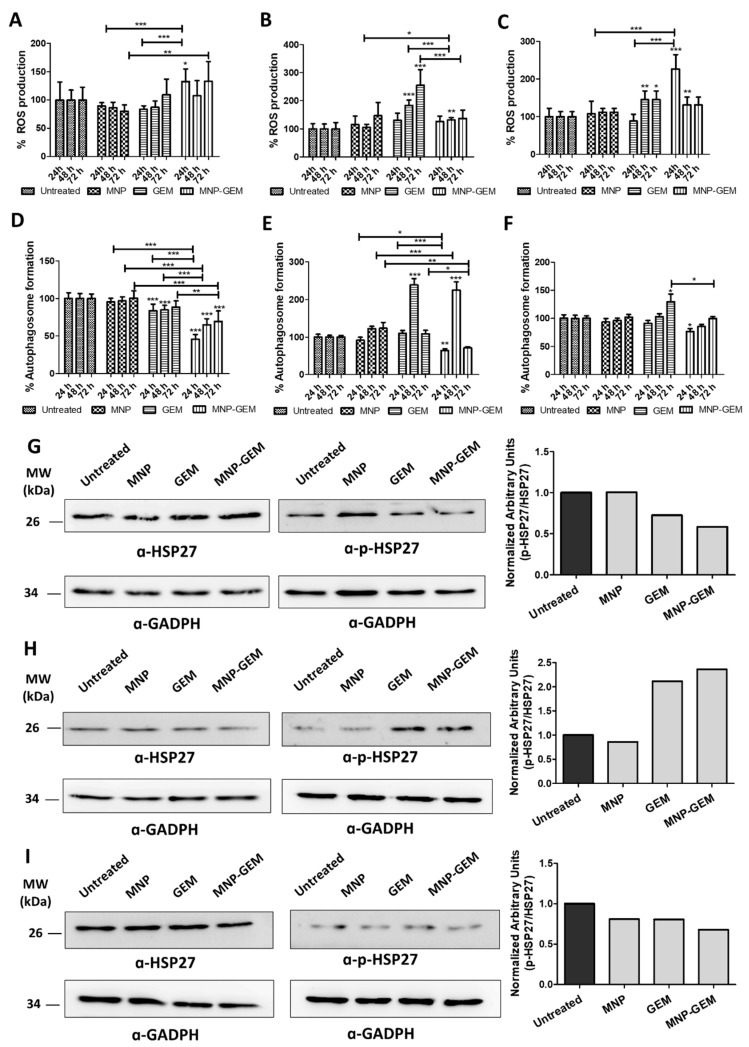
(**A**–**C**) Quantification of ROS levels by the detection of oxidized DCF-DA 24, 48, and 72 h after treatment; (**D**–**F**) Quantification of autophagosome formation by measuring the fluorescence of MDC 24, 48, and 72 h after treatment. Data of: (**A**,**D**) PANC-1; (**B**,**E**) BxPC-3; (**C**,**F**) MIA Paca-2. Conditions in PANC-1: MNP 0.5 mg Fe/mL, GEM 22.5 µM and MNP-GEM 0.5 mg Fe/mL 22.5 µM. Conditions in BxPC-3 and MIA Paca-2: MNP 0.1 mg Fe/mL, GEM 4.5 µM and MNP-GEM 0.1 mg Fe/mL 4.5 µM. Data represent mean ± SD. Statistical analysis was performed using one-way ANOVA test (each group vs. control). * *p* < 0.05, ** *p* < 0.001, *** *p* < 0.001. (**G**–**I**) Analysis of HSP-27 phosphorylation in PANC-1, BxPC-3, and MIA Paca-2, respectively. Densitometry analysis plots show arbitrary units calculated as a p-HSP-27 signal normalized to a HSP-27 signal. Before p-HSP-27/HSP-27 normalization, p-HSP-27 or HSP-27 signal was normalized to the GADPH signal.

We observed that MNP-GEM formulation slightly increased ROS production in PANC-1 cells, matching the same levels as GEM 72 h after treatment (Figure 5A). It is particularly interesting that ROS production mediated by MNP-GEM was higher than GEM 24 h and 48 h after treatment. However, in BxPC-3 cells, only GEM increased ROS production 48 h and 72 h after treatment (Figure 5B). In the case of MIA Paca-2 cells, MNP-GEM triggered an increase of ROS production after 24 h of treatment that was not observed with MNP and GEM separately. This increase in ROS production is maintained and reached equal limits in cells treated with GEM and MNP-GEM 48 h and 72 h after treatment (Figure 5C). Remarkably, MNP were not implicated in ROS production in these cell lines (Figure 5A–C).

### 3.9. Monodansylcadaverine Staining and Autophagosome Detection

We evaluated the effect of our systems in autophagy, which has several implications in cancer progression or suppression [41,44], and where nanoparticles have shown a remarkable effect [45].

In PANC-1 the reduction in autophagy was observed with GEM and, especially, with MNP-GEM (Figure 5D), the two conditions associated with cell death. But the opposite effect was found in the other cell lines, since these treatments induced an increase in autophagy activity 48 h after treatment in BxPC-3 (Figure 5E), and only GEM increases autophagosome formation in MIA Paca-2 after 72 h (Figure 5F). 

### 3.10. Analysis of HSP27 Phosphorylation in Gemcitabine Treated Pancreatic Cancer Cells

Heat shock proteins (HSP) can modify the function of key components of the apoptotic signaling pathway [46]. Particularly, HSP27 phosphorylation is crucial in the response to GEM in pancreatic cancer [47,48]. Herein, we analyzed this molecular mechanism in pancreatic cancer cells treated with MNP-GEM. In the case of PANC-1, the p-HSP27/HSP27 ratio was downregulated when cells are treated with GEM and MNP-GEM (Figure 5G). The same effect was observed in MIA Paca-2, although that reduction was only clear when cells were treated with MNP-GEM (Figure 5H). On the contrary, in BxPC-3, a significant increase occurred when cells were treated with GEM or MNP-GEM (Figure 5I). 

### 3.11. Necrosis/Apoptosis Assay

The two traditional cell death mechanisms, apoptosis and necrosis, were studied. On the one hand, in PANC-1 and BxPC-3 a slight increase in late apoptotic or necrotic cells was observed in GEM and MNP-GEM conditions in comparison with MNP and untreated cells (Figure S10A,B). On the other hand, MIA Paca-2 cells treated with GEM revealed a significant increase in early apoptosis, and late apoptosis or necrosis compared to the other conditions tested. Despite this, a minimal increase existed among late apoptotic cells in MNP-GEM (Figure S10C).

### 3.12. Magnetic Hyperthermia Evaluation in 2D Cell Cultures

**Figure 6:**
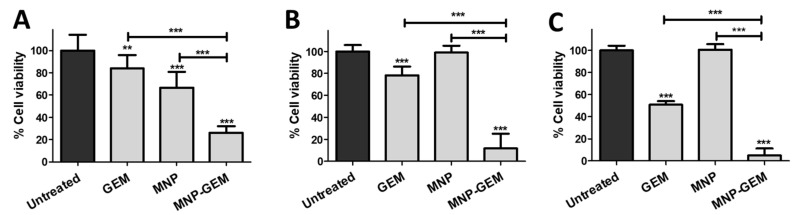
Cell viability assays in: (**A**) PANC-1; (**B**) BxPC-3; (**C**) MIA Paca-2 48 h after AMF (202 MHz, 29.9 mT, 20 min) was applied. Conditions in PANC-1: MNP 0.5 mg Fe/mL, GEM 22.5 µM, and MNP-GEM 0.5 mg Fe/mL 22.5 µM. Conditions in BxPC-3 and MIA Paca-2: MNP 0.1 mg Fe/mL, GEM 4.5 µM and MNP-GEM 0.1 mg Fe/mL 4.5 µM. Data represent means ± SD. Statistical analysis was performed using one-way ANOVA test (each group vs. control). ** *p* < 0.01, *** *p* < 0.001.

Finally, we evaluated the combined effect of MH and GEM in 2D cell cultures of PANC-1 (MNP 0.5 mg Fe/mL, GEM 22.5 µM and MNP-GEM 0.5 mg Fe/mL 22.5 µM), BxPC-3 (MNP 0.1 mg Fe/mL, GEM 4.5 µM and MNP-GEM 0.1 mg Fe/mL 4.5 µM) and MIA Paca-2 cells (MNP 0.1 mg Fe/mL, GEM 4.5 µM and MNP-GEM 0.1 mg Fe/mL 4.5 µM) (Figure 6 and Figure S11).

In PANC-1 cells, there were no statistical differences in cell viability among cells treated with MNP and MNP-GEM 24 h after the AMF (202 MHz, 29.9 mT, 20 min) was applied (Figure S11A). At this time, the reduction of cell viability of MNP-GEM was mainly produced by MH, because a similar reduction was achieved by MNP. However, 24 h later, the reduction in viability was 220% higher in the case of MNP-GEM than in MNP in the presence of an AMF. By comparing the cytotoxic effect of MNP-GEM, we observed that it is 173% higher in the presence of an AMF with MNP-GEM formulation in comparison with no HT treatment (Figure 2B). In BxPC-3 and MIA Paca-2 cells, there was no effect due to MH using MNP after 24 h, and the cytotoxic effect of MNP-GEM was related mainly with the drug (Figure S11B,C). Particularly, the reduction in cell viability was 270% and 138% higher in the presence of MNP-GEM and an AMF in BxPC-3 and MIA Paca-2, respectively (Figure 6B,C), in comparison with no HT treatment (Figure 2D,E).

## 4. Discussion

We have synthesized γ-Fe_2_O_3_ nanoparticles (Figure 1A,B) coated with dextran (MNP). They are a highly homogeneous formulation with a proper size for tumor cell uptake [49], since MNP size is around 100 nm and a PDI < 0.2 (Figure 1C), and they also present superparamagnetic properties (Figure 1D). Then, MNP were successfully funcitonalized with GEM (Figure 1E–G), presenting good stability even in cell culture media (Figure S2). The change in the Z-potential of MNP (−12.3 mV) was notable when they were modified by the drug (+15.1 mV), which suggests the successful modification of MNP. It is worth mentioning that the presence of disulfide bonds between MNP and GEM in our nanocarrier permitted the controlled release of the drug without any chemical modification (Figure S3A) and with high selectivity due to the reducing conditions present in the tumor (Figure S3B) [20,21]. Hence, our proposed nanocarrier offers a potential solution to avoid the toxicity and the lack of effectiveness of gemcitabine in its clinical use [3,4] and it is expected to remain intact in blood circulation [50]. The stability and efficacy of disulfide-based drug delivery systems have been widely studied, showing promising results in breast cancer [51], uveal melanoma [52], and pancreatic cancer models [19], among others. 

Moreover, the results suggest a reduced interaction between our nanoparticles and albumin, especially with MNP-GEM (Table 1, Figure S4), but the presence of GEM does not affect the nature of the interactions (Table S2). This is one of the main concerns on the use of nanoparticles in vivo, since this process modulates the pharmacokinetics of the nanoparticles and, therefore, can interfere with their safety and efficacy [53,54]. Furthermore,, the heat generated by MNP and MNP-GEM was studied to address their therapeutic potential in combination with magnetic hyperthermia. The high capacity of producing heat by our formulations was confirmed even in cell culture media (Table 2, and Figure S5). Thus, a high potential is expected for MNP-GEM in terms of biosafety for the limited interactions with plasma proteins [55] and for hyperthermia applications for the preservation of their magnetic properties [56]. The cell viability studies in cancer cells lines proved a remarkable cytotoxic effect of MNP-GEM, especially relevant in the gemcitabine-resistant pancreatic cancer cells PANC-1 (Figure 2). Moreover, MNP presented negligible toxicity, highlighting the good biocompatibility of this nanostructure. Therefore, this nanodevice can be employed to increase the efficacy of therapies against both sensitive and resistant pancreatic cancer cell lines and be more selective, since the drug will be better released at the target cells due to the sensitive linker used. Additionally, MNP-GEM were also effective in the breast cancer cell line MCF-7, and, more importantly, they were less toxic than the free drug in non-tumoral cells, probably due to the reduced release of the drug under non-tumoral conditions (Figure S7). These results are in agreement with the release studies done in vitro (Figure S3). It is worth noting that the cytotoxic effect of GEM is better observed 48 h (Figures S6 and S7) and 72 h (Figure 2 and Figure S7) after treatment, although the drug is completely released after 8 h in tumoral conditions (Figure S3). This can be explained by the mechanism of action of GEM. It is a prodrug that must be metabolized to the active triphosphate form in the cytoplasm. Then, it needs to reach the nucleus to inhibit the DNA synthesis by preventing chain elongation once it is incorporated into the DNA [57,58]. Thus, the reduction of cell viability will only be observed some time after incubation.

Moreover, the results of cell cycle analysis (Figure 3) are in concordance with the reported information for GEM. Thus, an arrest in the G1/S or S phase boundary was expected due to the mechanism of action of GEM, which is a DNA replication stress inducer that triggers senescence in cells [58,59]. On the other hand, the lack of modifications in the cell cycle due to the treatment of MNP confirmed that the effect of MNP-GEM is due to the presence of the drug in the nanocarrier. 

Interestingly, the internalization studies suggest that the changes in the MNP surface due to the presence of GEM noticeably enhanced the internalization of the nanocarrier in the pancreatic cancer cell lines tested (Figure 4 and Figure S8), but its presence did not affect the endocytic pathways involved in their internalization (Figure S9). There are two possible explanations of this effect. Firstly, it could be related to the more positively charged surface of MNP-GEM, which is known to improve interactions with negatively charged cell membranes [60]. Secondly, MNP-GEM can interact with specific receptors that this drug uses for being internalized [61]. Taking all into consideration, the novelty of our system resides in the remarkable efficacy of MNP-GEM compared to other gemcitabine delivery systems previously reported that also need targeting moieties such as antibodies [18] or peptides [19] to present high internalization and cytotoxic effect.

To further assess the cell death mechanism of MNP-GEM formulations, ROS and autophagy implications were studied (Figure 5) at three different time points (24, 48, and 72 h after treatment) to evaluate their tendency Regarding the role of ROS, its high production in PANC-1 cells treated with MNP-GEM in comparison with GEM could explain the better efficacy of our formulation in this cell line (Figure 5A). Nevertheless, in BxPC-3 cells, the ROS input produced by GEM was not observed with MNP-GEM (Figure 5B), suggesting that the mechanisms involved in drug transportation by MNP-GEM interfere with the production of ROS by this cytotoxic agent. By contrary, in MIA Paca-2 cells, an input in ROS production by MNP-GEM (Figure 5C) is not translated into a more cytotoxic effect than GEM. Therefore, we can conclude that the cytotoxic effect observed with MNP-GEM might be related to ROS exacerbation in PANC-1 and MIA Paca-2 but not in BxPC-3 cells. In respect of autophagy, the results suggest that it may have a protective role in PANC-1 (Figure 5D) and a pro-cell-death mechanism in BxPC-3 (Figure 5E), since it is only affected by the treatments associated with cell death. However, its role in MIA Paca-2 is not that clear since the increase in autophagosome formation triggered by GEM did not lead to a higher cytotoxic effect compared to MNP-GEM (Figure 5F). Additionally, the phosphorylation of HSP27 (p-HSP27) was also assessed since it has demonstrated to play an important role in the response to gemcitabine treatment in pancreatic cancer [47,48]. Our studies suggest that that p-HSP27/HSP27 proportion due to GEM treatment depends on the cell line tested (Figure 5G–I). Similar effects are observed between the drug alone and our proposed nanocarrier, suggesting that the cytotoxic effect of the drug is conserved or even improved with our nanocarrier.

According to necrosis/apoptosis studies, in PANC-1 and BxPC-3, a slight increase of late apoptotic or necrotic cells in GEM and MNP-GEM conditions (Figure S10A,B) which is in concordance with the mechanism of action expected by GEM [62]. However, the differences with the untreated cells are not as much as it was expected. For this reason, a clear mechanism of cell death cannot be deduced. However, in MIA Paca-2 there was an apparent increase in apoptotic and necrotic cells when they were treated with GEM but no with MNP-GEM (Figure S10C), suggesting a different cell death mechanism for MNP-GEM. The observed differences in cell death mechanism observed for GEM in comparison with MNP-GEM are not surprising, since the release mechanism of the drug from the nanocarrier might have an effect on the mechanism of action [63].

Finally, the results of the cell viability studies in combination with MH demonstrate that the cytotoxic effect of MNP-GEM can be enhanced in the presence of an AMF. It is widely known that MH can produce a direct cytotoxic effect and sensitize tumor cells to respond favorably to chemotherapy [31] and its cytotoxic effect is commonly observed when tumoral cells reach 40–46 °C [64,65]. In this sense, MNP formulation produced a cytotoxic effect in PANC-1 and no in BxPC-3 and MIA Paca-2 cells, which is consistent with the data obtained from hyperthermia assays in solution (Table 2 and Figure S5) since only the concentration tested in the gemcitabine-resistant cells can reach higher temperatures than 40 °C for MNP and MNP-GEM. However, it has also been reported that cell death produced by MH can occur without a noticeable global increase in temperature in the medium containing cells [66,67]. This scenario happens in BxPC-3 and MIA Paca-2 cells, according to MH data in solution and can be related to the effect observed by MNP-GEM (Table 2 and Figure S5). But what is more interesting is the synergistic effect observed after the AMF was applied (Figure 6). This excellent antitumoral activity could be due to the increased membrane fluidity due to MH and passive uptake of the diffusing species [68]. It is possible that the alterations in membrane fluidity allowed the internalization of more MNP-GEM, leading to a higher cytotoxic effect. Also, MNP-GEM are better internalized by cells than MNP (Figure 4B and Figure S9B,D). Thus, the intracellular contribution of MH is magnified when cells are treated with MNP-GEM.

Considering all the information reported, we will expect advantages to using MNP-GEM in comparison with GEM in an in vivo pancreatic cancer model. There are reports in the literature where MNP have been tested in vivo in subcutaneous and xenograft mouse models of pancreatic cancer [13,14,19,69,70]. In the studies wherein the nanoparticles are intratumorally injected [13,14,19], the results highlight the good biocompatibility of iron-oxide-based formulations and the efficacy of hyperthermia treatment with high loads of the particles. However, it is also common to observe the biodistribution of the particles also in the liver and spleen. Although this is the simplest model to test the potential of MNP in a solid tumor such as pancreatic cancer, some works have tried to test the efficacy of their magnetic nanoparticles-based systems via intravenous injection [67,68]. In these cases, it is a more realistic model, and the main advantage is that MNP can also be used as a diagnostic tool since they can be seen in MRI [10]. In the present study, MNP-GEM present an appropriate size to reach the tumor site through enhanced permeability and retention (EPR) effect [49,71] and have demonstrated good biocompatibility with plasma proteins and non-tumoral cells, which make them also suitable for intravenous injection [72]. Moreover, the comparison of MNP and MNP-GEM in terms of internalization would be hugely interesting, since an increased uptake of MNP-GEM would be probable, considering our cell culture experiments. Nevertheless, the effects of MNP-GEM would be observed some time after treatment due to the mechanism of action of the drug and the release mechanism, mentioned above. What is more, an exceptional cytotoxic effect would be possible in combination with MH.

## 5. Conclusions

We have successfully functionalized MNP with GEM (MNP-GEM) via disulfide bonds. MNP-GEM is a smart drug delivery system designed to obtain a highly selective release of the drug in pancreatic cancer cells. Remarkably, the changes in the surface associated with the functionalization of MNP with the drug reduced the binding of plasma proteins and dramatically enhanced the internalization of the nanocarrier in all cancer cells tested. Our experiments demonstrated that our nanoparticles are stable in biological media and can be used for MH. Moreover, the cell viability assays revealed that MNP-GEM had significant cytotoxicity against different pancreatic cell lines and its cytotoxic effect seems to be related with the cell cycle arrest that occurs in G1-S phase as well as the role of p-HSP27. Additionally, it is less toxic for non-tumoral cells, suggesting a high selectivity against cancer. Interestingly, this nanocarrier is particularly efficient in PANC-1 cells, the most resistant to GEM, where an increase in ROS production and a decrease in autophagosome formation seem to be closely related to cell death mechanisms. What is more, the toxicity of MNP-GEM increases when an AMF is applied in all pancreatic cell lines tested, even in the most resistant to the drug.

## Figures and Tables

**Table 1:** The quenching constants of albumin by MNP and MNP-GEM at two different temperatures.

Albumin-Nanoparticle Complex	Temperature [K]	Stern–Volmer Quenching Constant k_sv_ [M^−1^]	Biomolecular Quenching Constant k_q_ [M^−1^·s^−1^]
Albumin-MNP	299.65	1.088 × 10^4^	1.844 × 10^12^
	310.15	1.001 × 10^4^	1.697 × 10^12^
Albumin-MNP-GEM	299.65	9.153 × 10^3^	1.634 × 10^12^
	310.15	8.884 × 10^3^	1.499 × 10^12^

**Table 2:** SAR and maximum temperature values of MNP and MNP-GEM in different conditions after an AMF was applied (202 kHz, 29.9 mT, 20 min).

Nanoparticles [0.5 mg Fe/mL]	Medium	SAR [W/g Fe]	Max. Temperature [°C]	Nanoparticles [0.1 mg Fe/mL]	Medium	SAR [W/g Fe]	Max. Temperature [°C]
MNP	Water	162.378	43.91	MNP	Water	401.760	39.16
	DMEM	110.484	40.37		DMEM	322.245	38.84
	RPMI	140.616	41.57		RPMI	217.620	37.95
MNP-GEM	Water	173.259	41.74	MNP-GEM	Water	322.245	39.34
	DMEM	103.788	43.80		DMEM	297.620	38.58
	RPMI	154.845	44.14		RPMI	309.690	38.98

## Data Availability

The data presented in this study are available on request from the corresponding author.

## Supplementary Materials

The following are available online at https://www.mdpi.com/article/10.3390/cancers13164095/s1, Materials for MNP synthesis, functionalization and characterization, Materials for cell culture studies, Table S1: Endocytosis inhibitors used to inhibit the uptake of nanoparticles, Table S2: Thermodynamic parameters for the interactions of albumin with MNP and MNP-GEM. Figure S1: General scheme of synthesis of gemcitabine derivative, Figure S2: Hydrodynamic size evaluation of MNP and MNP-GEM in water, PBS, and DMEM for seven days, Figure S3: Gemcitabine release from MNP-GEM, Figure S4: Stern–Volmer plots of albumin with increasing concentrations of MNP or MNP-GEM nanoparticles at two different temperatures (26.5 °C and 37 °C), Figure S5: Evaluation of magnetic hyperthermia produced by MNP and MNP-GEM, Figure S6: Cell viability assays in PANC-1, BxPC-3, and MiaPaca-2 48 h after treatment, Figure S7: Cell viability assays in HaCaT cells 48 h and 72 h after treatment, Figure S8: Internalization study of MNP and MNP-GEM in BxPC-3 and MiaPaca-2, Figure S9: Nanoparticles internalization in PANC-1, BxPC-3, and MiaPaca-2 in the presence of inhibitors of endocytic pathways, Figure S10: Flow cytometry analysis of apoptosis/necrosis 48 h after treatment in PANC-1, BxPC-3, and MIA Paca-2 cells, Figure S11: Cell viability assays in PANC-1, BxPC-3, and MIA Paca-2 24h after AMF was applied. Figure S12. Calibration line of gemcitabine in PBS. Absorbance measurements at 270 nm in a Synergy H4 microplate reader.
