# Supplementary material for: Smart Modification on Magnetic Nanoparticles Dramatically Enhances Their Therapeutic Properties
Source: Cancers (Basel). 2021 Aug 14;13(16):4095. doi: 10.3390/cancers13164095 (PMC8391586; doi:10.3390/cancers13164095)
Supplement: Supplementary file 1 [file cancers-13-04095-s001.zip › cancers-1329849-supplementary/cancers-1329849-supplementary.pdf]

cancers

MDPI

Smart Modification on Magnetic Nanoparticles Dramatically Enhances their Therapeutic Properties

Nuria Lafuente-Gómez, Paula Milán-Rois, David García-Soriano, Yurena Luengo, Marco Cordani, Hernán Alarcón-Iniesta, Gorka Salas and Álvaro Somoza *

Supplementary Materials

Materials for MNP Synthesis, Functionalization and Characterization

$FeCl_2$, $FeCl_3$, NH_4OH 25%, $Fe(NO_3)_3$, HNO_3, NaOH and Dextran 40 KDa were purchased in Sigma Aldrich. Ultrapure reagent grade water MiliQ (18,2 MΩ, Wasserlab) was used in all experiments. For MNP functionalization, gemcitabine derivative (Gem-S-S-Pyr) was prepared according to described procedures. Gemcitabine was purchased from Fluorochem. Aldrithiol, 2-mercaptoethanol, bis(4-nitrophenylcarbonate), N,N-diisopropylehylamine (DIPEA), 4-(dimethylamine)pyridine (DMAP), 1-ethyl-3-(3-dimethylaminopropyl)carbodiimide) (EDC), N-hydroxysuccinimide (NHS), cysteamine hydrochloride and sodium hydroxide (NaOH), Bovine serum albumin (BSA) and 1,4-dithiothreitol (DTT) were purchased from Sigma Aldrich. The solvents dichloromethane (CH_2Cl_2), hexane, ethyl acetate (AcOEt), methanol (MeOH), and N,N′-dimethylformamide (DMF) were purchased from Scharlab.

Materials for Cell Culture Studies

Modified Eagle's medium (DMEM), streptomycin–penicillin 10X, Roswell Park Memorial Institute (RPMI) medium, fetal bovine serum (FBS), L-glutamine 10X, trypsin 1X, phosphate-buffered saline (PBS), and cell culture plasticware were purchased from VWR. Dimethyl sulfoxide (DMSO), resazurin sodium salt, propidium iodide (PI), RNAasa A, potassium ferrocyanide trihydrate, hydrochloric acid (HCl), neutral red, ferrozine, ascorbic acid, ammonium acetate, neocuproine, potassium permanganate ($KMnO_4$), paraphormaldehyde, glutaraldehyde, chlorpromazine, genistein, filipin III, colchicine, cytochalasin D, sodium azide, Diacetylated 2,7 -dichlorofluorescein (DCF-DA) and monodansylcadaverine (MDC), Tris-HCl, EDTA, sodium chloride, glycerol, Triton X-100, sodium fluoride, sodium pyrophosphate, sodium orthovanadate, phenylmethylsulfonyl, and Tween 20 were obtained from Sigma Aldrich. Ethanol (EtOH) was purchased from Scharlab. The antibodies used for western-blot analysis anti-cyclin E (sc-377100), anti-HSP27 (sc-13132), anti-phospho HSP27 (sc-166693), anti-GADPH (sc-47724) and peroxidase-labelled anti-mouse (sc-516102) were purchased in Santa Cruz Biotecnhology.

PANC-1 and MCF-7 cells were obtained from ATCC, BxPC-3 and MIA Paca-2 were kindly provided from Ibane Abasolo's group of Vall d'Hebron Institut de Recerca (VHIR), and HaCaT were kindly provided by Pilar Martín Duque from Universidad Francisco de Vitoria.

PANC-1, MIA Paca-2, MCF-7 and HaCaT cells were cultured in DMEM medium and BxPC-3 cells were cultured in RPMI medium. Both cell culture media were supplemented with 10% FBS, 1% streptomycin–penicillin and 1% L-glutamine and cells were incubated at 37 °C in a Binder CB210 incubator (5% CO_2). All the procedures were performed inside a laminar flow hood Telstar CV-30/70.

Equations for the Evaluation of Protein Binding

The interaction of the MNP with albumin protein was analyzed by studying the possible quenching mechanism of BSA using the Stern-Volmer equation (equation S1) [1–3].

$$\frac{F_0}{F} = 1 + k_q \tau_0 [Q] = 1 + k_{sv}[Q] \qquad \text{(S1)}$$

Where F_0 is the fluorescence intensity in the absence of MNP or MNP-GEM, F is the fluorescence intensity in the presence of MNP or MNP-GEM, τ_0 is the lifetime of the BSA (5.9 ns) in the absence of MNP or MNP-GEM, $[Q]$ is the concentration of MNP or MNP-GEM, k_{sv} is the Stern-Volmer quenching constant, and k_q is the bimolecular quenching constant.

The nature of the binding force that exists between BSA and magnetic nanoparticles was determined by evaluating the thermodynamic parameters using the Van't Hoff equation (equation S2). This equation can be used when the enthalpy changes do not much variate over the temperature ranges studied [1,2].

$$\ln\left(\frac{k_{b\,(T_2)}}{k_{b\,(T_1)}}\right) = \frac{\Delta H\left(\frac{1}{T_1}-\frac{1}{T_2}\right)}{R} \quad \text{(S2)}$$

Where $k_{b\,(T_1)}$ and $k_{b\,(T_2)}$ are the binding constants at T_1 (299.65 K) and T_2 (310.15 K), respectively, ΔH is the change in enthalpy (J mol^{-1}) and R is the universal gas constant (8.3145 J·mol^{-1}·K^{-1}). To determine the change in Gibb's energy (ΔG) and entropy (ΔS) values, we used the thermodynamic equations S3 and S4 [1].

$$\Delta G(T) = -RT \ln K_{b(T)} \quad \text{(S3)}$$

$$\Delta S(T) = -\frac{\Delta G - \Delta H}{T} \quad \text{(S4)}$$

Table S1. Endocytosis inhibitors used to inhibit the uptake of nanoparticles.

Compound	Uptake Mechanism Inhibited	Concentration [μg/mL]
Genistein	Caveolin-mediated endocytosis	36
Filipin III	Caveolin-mediated endocytosis	5
Chlorpromazine	Clathrin-mediated endocytosis	10
Colchicine	Macropinocytosis	8
Cytochalasin D	Actin-mediated endocytosis	2.5
	Macropinocytosis and phagocytosis	
Sodium azide	ATP-mediated uptake	3

Table S2. Thermodynamic Parameters (ΔH, ΔG and ΔS) for the interactions of albumin with MNP and MNP-GEM at 26.5 °C (299.15 K) and 37 °C (310.15 K).

Albumin-nanoparticle Complex	Temperature [K]	ΔH [kJ·mol^{-1}]	ΔG [kJ·mol^{-1}]	ΔS [kJ·mol^{-1}·K^{-1}]	Nature of the Binding Force
albumin-MNP	299.65	−6.133	−23.157	0.0568	ΔH<0, ΔS>0 Electrostatic interaction
	310.15		−23.753	0.0568	ΔH<0, ΔS>0 Electrostatic interaction
albumin-MNP-GEM	299.65	-2.191	−22.726	0.06853	ΔH<0, ΔS>0 Electrostatic interaction
	310.15		−23.446	0.06853	ΔH<0, ΔS>0 Electrostatic interaction

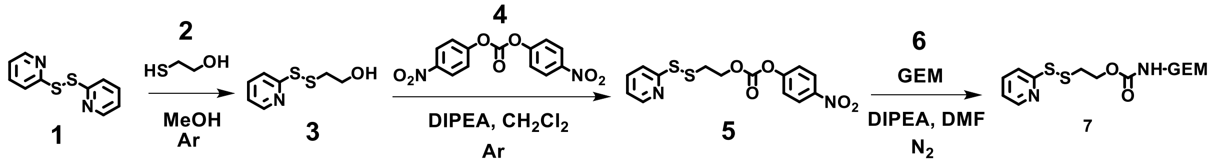

Figure S1. General scheme of synthesis of gemcitabine derivative according to a described procedure [4]. 1.- Aldrithiol, 2.- 2-Mercaptoethanol, 3.- 2-(pyridine-2-yldisulfanyl)ethanol, 4.-bis(4-nitrophenyl)carbonate, 5.-4-nitrophenyl 2-(pyridine-2-yldisulfanyl)ethyl carbonate, 6.- Gemcitabine, 7.-GEM-s-s-Pyr.

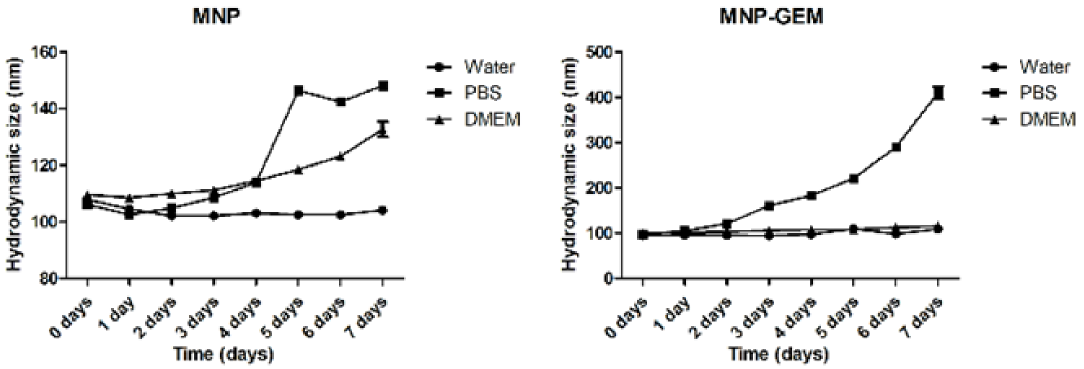

Figure S2. Hydrodynamic size evaluation of MNP and MNP-GEM in water, PBS and DMEM supplemented with 10% of FBS for seven days.

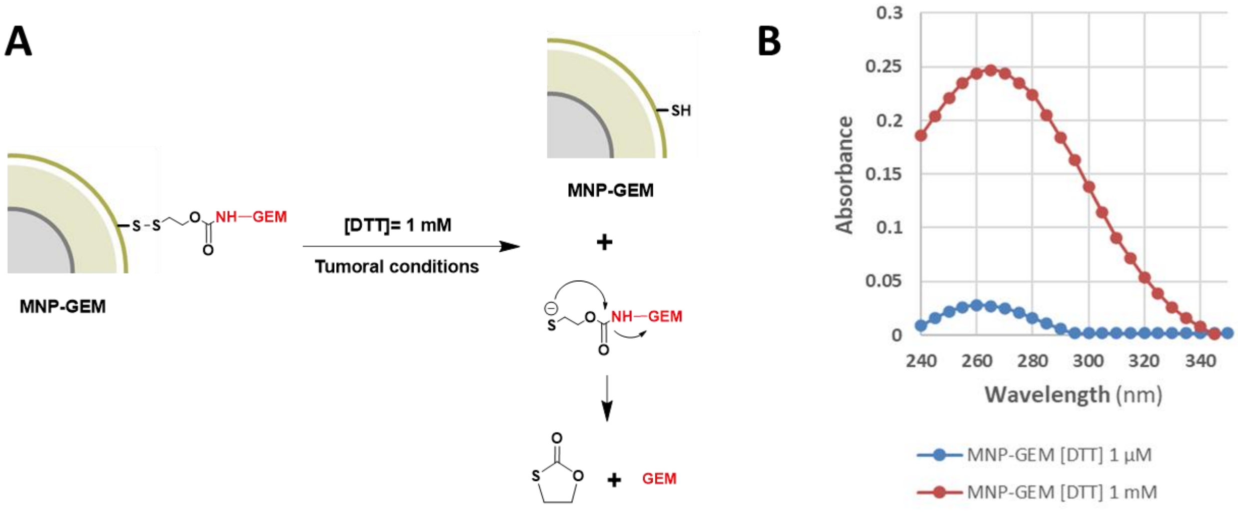

Figure S3. Gemcitabine release from MNP-GEM. **(A)** Scheme of gemcitabine release from MNP-GEM in tumoral conditions. **(B)** Spectrum of the release of GEM from MNP-GEM after 8 h being in tumoral mimicking conditions (MNP-GEM DTT 1 mM) and healthy tissues conditions (MNP-GEM DTT 1 µM).

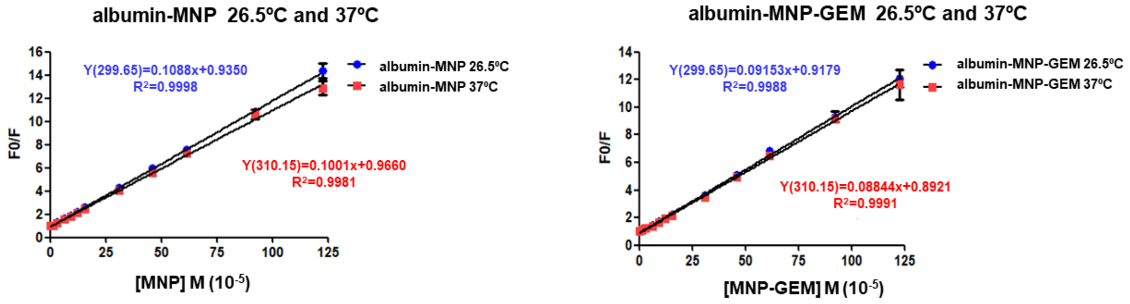

Figure S4. Stern-Volmer plots of albumin with increasing concentrations of MNP or MNP-GEM nanoparticles at two different temperatures (26.5 °C and 37 °C).

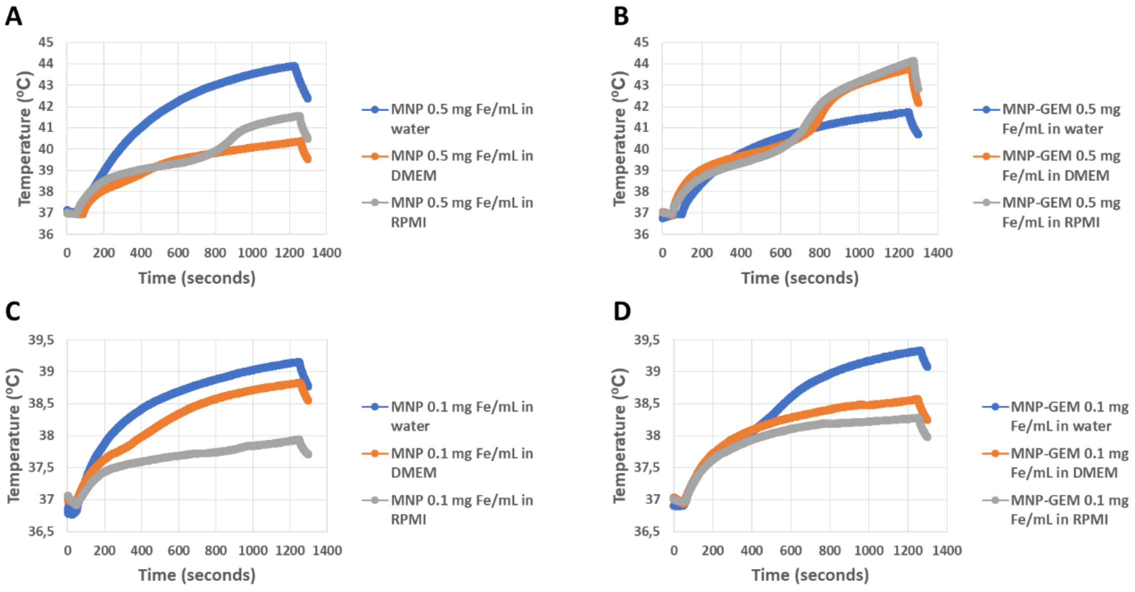

Figure S5. Evaluation of magnetic hyperthermia produced by: **(A)** MNP at 0.5 mg Fe/mL; **(B)** MNP-GEM at 0.5 mg Fe/mL; **(C)** MNP 0.1 mg Fe/mL; **(D)** MNP-GEM and 0.1 mg Fe/mL. AMF applied for 20 minutes at 202 kHz and 29.9 mT.

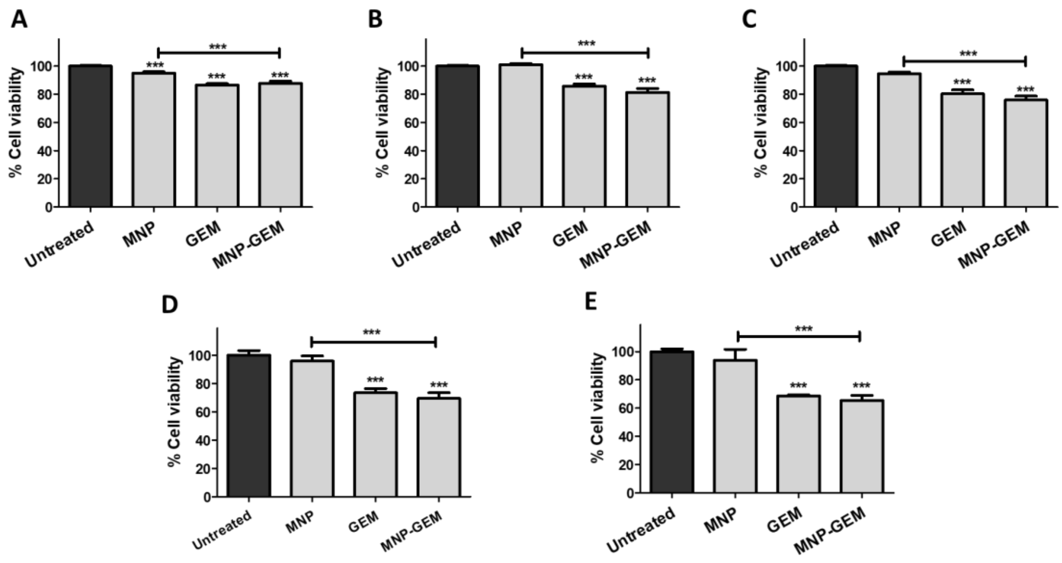

Figure S6. Cell viability assays in: **(A), (B), (C)** PANC-1; **(D)** BxPC-3; **(E)** MIA Paca-2 cells 48 h after treatment. Conditions tested: **(A), (D), (E)**: MNP 0.1 mg Fe/mL, GEM 4.5 μM, MNP-GEM 0.1 mg Fe/mL 4.5 μM; **(B)** MNP 0.5 mg Fe/mL, GEM 22.5 μM, MNP-GEM 0.5 mg Fe/mL 22.5 μM; **(C)** MNP 2 mg Fe/mL, GEM 90 μM, MNP-GEM 2 mg Fe/mL 90 μM. Data represent means ± SD. Statistical analysis was performed using a one-way ANOVA test (each group vs. control). *** $p < 0.001$.

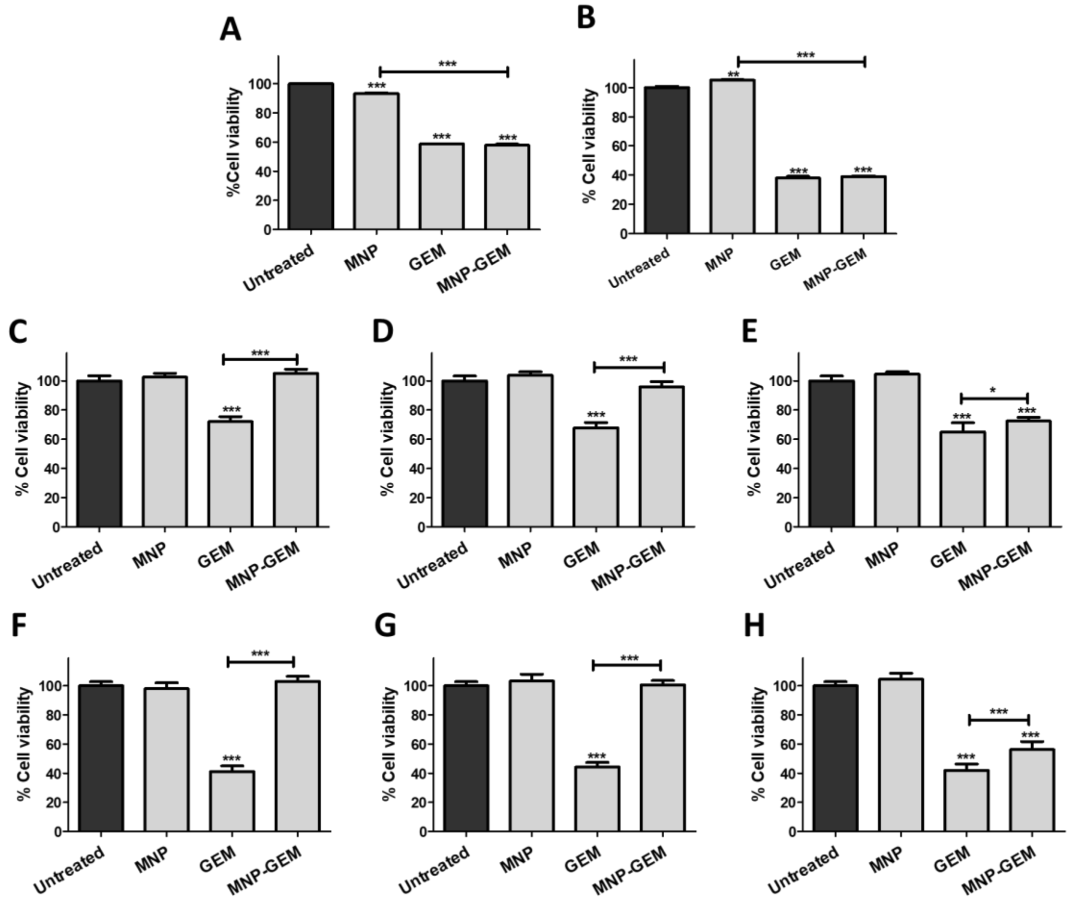

Figure S7. Cell viability assays in: **(A)**, **(B)** MCF-7 and **(C), (D), (E), (F), (G), (H)** HaCaT cells. **(A), (C), (D), (E)** 48 h after treatment; **(F), (G), (H)** 72 h after treatment. Conditions tested. **(a), (c), (f):** MNP 0.1 mg Fe/mL, GEM 4.5 μM, MNP-GEM 0.1 mg Fe/mL 4.5 μM. **(D), (G)**: MNP 0.5 mg Fe/mL, GEM 4.5 μM, MNP-GEM 0.5 mg Fe/mL 4.5 μM. **(E)**, **(H)**: MNP 2 mg Fe/mL, GEM 90 μM, MNP-GEM 2 mg Fe/mL 90 μM Statistical analysis was performed using one-way ANOVA test (each group vs control). * $p < 0.05$, ** $p < 0.001$, *** $p < 0.001$.

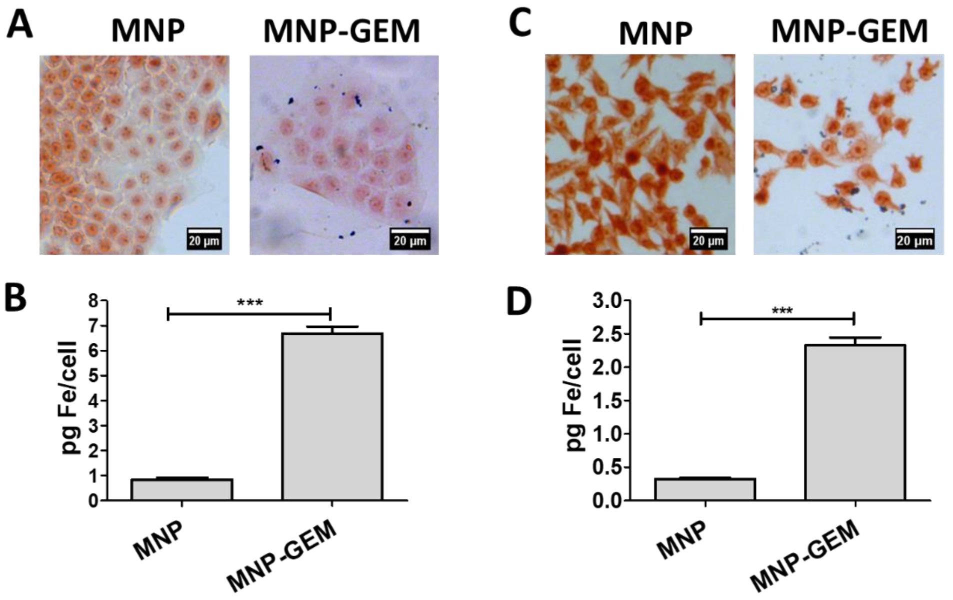

Figure S8. Internalization study of MNP and MNP-GEM in: **(A), (B)** BxPC-3; **(C), (D)** MIA Paca-2 at 0.1 mg Fe/ml. **(A), (C)** Prussian blue staining; **(B), (D)** ferrozine assay. Data represent means ± SD. Statistical analysis was performed using Student's *t*-test (MNP vs. MNP-GEM). *** $p < 0.001$.

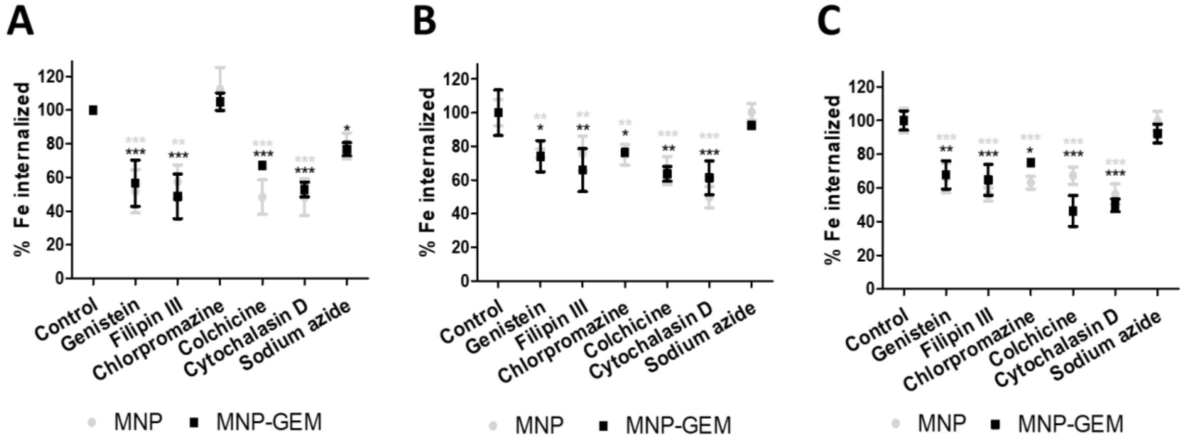

Figure S9. Nanoparticles internalization in: **(A)** PANC-1; **(B)** BxPC-3; **(C)** MIA Paca-2 in the presence of Inhibitors of endocytic pathways. Data represent means ± SD. Statistical analysis was performed using one-way ANOVA test (control vs each inhibitor). * $p < 0.05$, ** $p < 0.001$, *** $p < 0.001$.

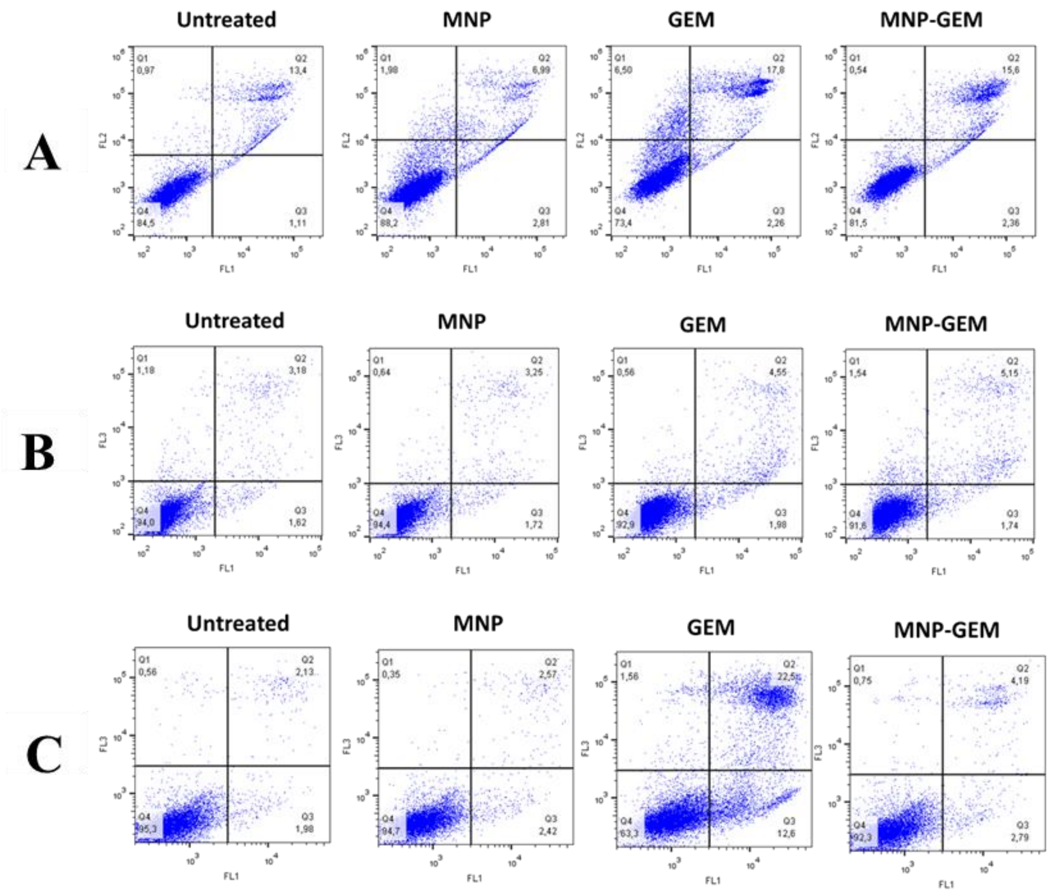

Figure S10. Flow cytometry analysis of apoptosis/necrosis 48 h after treatment in: **(A)** PANC-1; **(B)** BxPC-3; **(C)** MIA Paca-2. Q1: % death cells due to trypsinization; Q2: % apoptotic cells; Q3: % necrotic cells; Q4: % alive cells. Conditions in PANC-1 cells: MNP 0.5 mg Fe/mL, GEM 22.5 μM and MNP-GEM 0.5 mg Fe/mL 22.5 μM. Conditions in MIA Paca-2 and BxPC-3 cells: MNP 0.1 mg Fe/mL, GEM 4.5 μM and MNP-GEM 0.1 mg Fe/mL 4.5 μM.

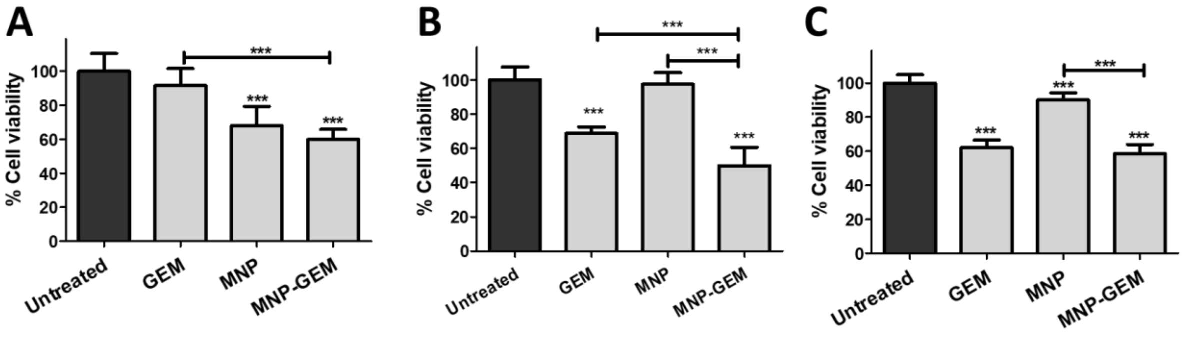

Figure S11. Cell viability assays in: **(A)** PANC-1; **(B)** BxPC-3; **(C)** MIA Paca-2 24 h after AMF (202 MHz, 29.9 mT, 20 min) was applied. Conditions in PANC-1: MNP 0.5 mg Fe/mL, GEM 22.5 μM and MNP-GEM 0.5 mg Fe/mL 22.5 μM. Conditions in BxPC-3 and MIA Paca-2: MNP 0.1 mg Fe/mL, GEM 4.5 μM and MNP-GEM 0.1 mg Fe/mL 4.5 μM. Data represent means ± SD. Statistical analysis was performed using one-way ANOVA test (each group vs. control). *** $p < 0.001$.

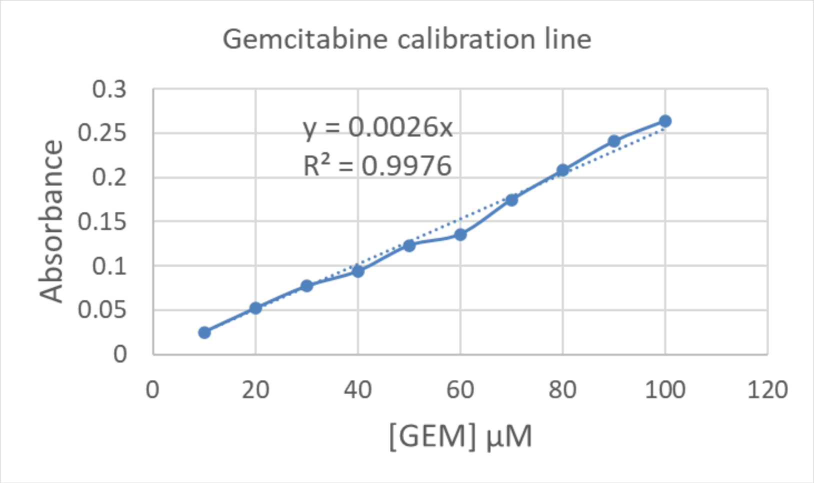

Figure S12. Calibration line of gemcitabine in PBS. Absorbance measurements at 270 nm in a Synergy H4 microplate reader.

References

1. Suvarna, M.; Dyawanapelly, S.; Kansara, B.; Dandekar, P.; Jain, R. Understanding the stability of nanoparticle-proteins interactions: Effect of particle size on adsorption, conformation and thermodynamic properties of serum albumin proteins. *ACS Appl. Nano Mater.* **2018**, *1*, 5524–5535, doi:10.1021/acsanm.8b01019.
2. Bekale, L.; Agudelo, D.; Tajmir-Riahi, H.A. Effect of polymer molecular weight on chitosan-protein interaction. *Colloids Surfaces B Biointerfaces* **2015**, *125*, 309–317, doi:10.1016/j.colsurfb.2014.11.037.
3. Lakowicz, J.R. *Principles of Fluorescence Spectroscopy.* 2006; ISBN 0-387-31278-1.
4. Latorre, A.; Couleaud, P.; Aires, A.; Cortajarena, A.L. Multifunctionalization of magnetic nanoparticles for controlled drug release: A general approach. *Eur. J. Med. Chem.* **2014**, *82*, 355–362, doi:10.1016/j.ejmech.2014.05.078.
